# CRISPR/Cas13 sgRNA‐Mediated RNA–RNA Interaction Mapping in Live Cells with APOBEC RNA Editing

**DOI:** 10.1002/advs.202409004

**Published:** 2024-10-11

**Authors:** Li‐Ting Diao, Shu‐Juan Xie, Wan‐Yi Xu, Hai‐Hui Zhang, Ya‐Rui Hou, Yan‐Xia Hu, Xin‐Xiang Liang, Jun‐Bin Liang, Qi Zhang, Zhen‐Dong Xiao

**Affiliations:** ^1^ Biotherapy Center, The Third Affiliated Hospital Sun Yat‐sen University Guangzhou 510630 P. R. China; ^2^ Institute of Vaccine The Third Affiliated Hospital Sun Yat‐sen University Guangzhou 510630 P. R. China; ^3^ Reforgene Medicine Guangzhou 510535 P. R. China; ^4^ Guangdong Provincial Key Laboratory of Liver Disease Research The Third Affiliated Hospital Sun Yat‐sen University Guangzhou 510630 P. R. China

**Keywords:** dCas13, lncRNA, proximal labeling, RNA editing, RNA‐RNA interaction

## Abstract

Current research on long non‐coding RNA (lncRNA) has predominantly focused on identifying their protein partners and genomic binding sites, leaving their RNA partners largely unknown. To address this gap, the study has developed a method called sarID (sgRNA scaffold assisted RNA‐RNA interaction detection), which integrates Cas13‐based RNA targeting, sgRNA engineering, and proximity RNA editing to investigate lncRNA‐RNA interactomes. By applying sarID to the lncRNA *NEAT1*, over one thousand previously unidentified binding transcripts are discovered. sarID is further expanded to investigate binders of *XIST*, *MALAT1, NBR2*, and *DANCR*, demonstrating its broad applicability in identifying lncRNA‐RNA interactions. The findings suggest that lncRNAs may regulate gene expression by interacting with mRNAs, expanding their roles beyond known functions as protein scaffolds, miRNA sponges, or guides for epigenetic modulators. sarID has the potential to be adapted for studying other specific RNAs, providing a novel immunoprecipitation‐free method for uncovering RNA partners and facilitating the exploration of the RNA‐RNA interactome.

## Graphical abstract

This study introduces a method called sarID for identifying lncRNA‐RNA interactions. The method uses dCas13b to target RNA and MS2 aptamers inserted into the tetraloop of the sgRNA to recruit APOBEC1, which labels the interacting transcripts with C‐to‐U editing in living cells. sarID represents a significant advancement in the understanding of RNA‐RNA interactions.

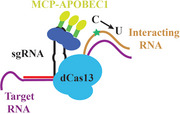

## 1 Introduction

lncRNAs outnumber protein coding genes in human genome and serve as important regulators of gene expression. Both in vitro and in vivo studies have demonstrated that numerous lncRNAs play crucial roles in various physiological and pathological processes.^[^ ^1^, ^2^, ^3^, ^4^, ^5^, ^6^ ^]^ These lncRNAs exert their effects on gene regulation through interactions with proteins, DNA, and RNA.^[^ ^7^, ^8^ ^]^ The functional diversity of lncRNAs is reflected in their complex mechanisms of action, which involve a wide range of interactions with cellular molecules.^[^ ^9^ ^]^ Some lncRNAs interact with chromatin, recruiting chromatin modifiers to activate or suppress transcription.^[^ ^10^ ^]^ Others, possessing protein interaction domains, act as scaffolds for proteins that are necessary for the formation and proper functioning of complexes.^[^ ^11^, ^12^, ^13^ ^]^ Additionally, certain lncRNAs interact with RNAs, such as those containing microRNA complementary sites, and function as sponges for microRNAs, thereby regulating the translation of mRNAs.^[^ ^14^ ^]^ However, there are still numerous novel molecular mechanisms of lncRNA action that have yet to be fully elucidated.

While current research on lncRNAs has focused primarily on identifying their protein partners and genomic binding sites, little is known about their RNA partners, especially RNAs other than miRNAs, largely due to the lack of efficient methodologies.^[^ ^15^ ^]^ Although recent biochemical crosslinking methods with subsequent high‐throughput sequencing have enabled the exploration of genome‐wide RNA‐RNA interactions, the low efficiency and sensitivity of these methods make them unsuitable for identifying the RNA‐RNA interactomes for specific lncRNAs.^[^ ^16^ ^]^ Established in vitro methods for identifying the partners of particular lncRNAs, such as RNA pulldown, have limitations such as interference from nonspecific associations and the use of in vitro‐transcribed (IVT) RNAs, which may not accurately reproduce the modifications and structure of the RNA of interest in cells,^[^ ^17^ ^]^ as bait. In vivo methods such as chromatin RNA immunoprecipitation (ChRIP) and RNA antisense purification (RAP) rely on crosslinking to facilitate efficient isolation, but crosslinking may introduce biases and obscure physiological interactions.^[^ ^18^, ^19^ ^]^ A recently developed hybridization proximity (HyPro)‐seq method uses the APEX2 enzyme to biotinylate RNAs colocalized with the transcript of interest but has limited application in vivo because of the high toxicity of H_2_O_2_.^[^ ^20^ ^]^ Furthermore, all existing in vitro and in vivo techniques utilize affinity capture of target RNAs, which can be labor intensive and may result in biases due to post‐lysis protein reassortment.^[^ ^21^ ^]^

In order to address these limitations and facilitate the identification of RNA partners for specific lncRNAs within living cells, we have developed a method called sarID. Initially, we first screened a truncated catalytically inactive pspCas13b to target lncRNA. Through examination of tagging positions, we discovered that inserting MS2 adapters into the sgRNA loop effectively recruited additional protein effectors to the Cas13‐sgRNA complex. Finally, by utilizing APOBEC1 for cytosine‐to‐uracil (C‐to‐U) editing, we successfully created a dCas13‐guided proximal labeling method known as sarID. We applied sarID to identify the transcript partners of *NEAT1*, *XIST*, *MALAT1, NBR2*, and *DANCR*, and found it to be highly efficient, revealing previously unidentified transcript partners for these specific lncRNAs. This suggests an unknown gene regulation paradigm in which lncRNAs interact with a large number of mRNAs. Although sarID was initially tested for lncRNAs, it can be easily adapted to identify transcript partners for other RNA species, greatly facilitating the exploration of the RNA‐RNA interactome within living cells.

## 2 Results

### 2.1 Assessment of the Binding Capacity of Catalytically Inactive Cas13 Orthologs

The Cas13 effector, a class 2 RNA‐guided RNA‐targeting CRISPR‐Cas system, can be modified for RNA knockdown.^[^ ^22^ ^]^ Interestingly, the catalytically inactive form (referred to as dead Cas13 or dCas13) retains its ability to bind to targeted RNA. And thus, dCas13 has been utilized for various in vivo RNA modifications, including programmable regulation of alternative splicing,^[^ ^23^, ^24^ ^]^ A‐to‐I and C‐to‐U editing,^[^ ^25^, ^26^ ^]^ m^6^A modifications,^[^ ^27^ ^]^ RNA‐protein interaction mapping,^[^ ^28^, ^29^, ^30^ ^]^ and RNA imaging.^[^ ^31^, ^32^ ^]^ As genome editing progresses rapidly, more and more Cas13 orthologs belonging to different subtypes have been discovered and characterized.

**Figure 1:**
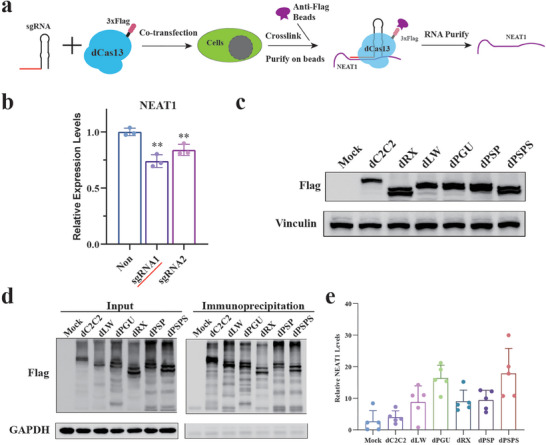
Assessment of the binding capacity of dCas13 orthologs. a) Schematics of the RIP assay. Catalytically inactive Cas13 ORFs fused with a 3xFlag peptide at the N‐terminus were co‐transfected with the corresponding sgRNAs targeting the same region of the *NEAT1* transcript. After crosslinking, RNP complexes were immunoprecipitated with anti‐Flag magnetic beads. b) Real‐time RT‐PCR measurement of *NEAT1* levels for assessing sgRNA efficiency. Two pspCas13b sgRNAs targeting *NEAT1* were predicted. The sgRNA1 targeting region was selected for further study. The data were presented as mean ± SDs, *n*  =  3 independent experiments, two‐tailed unpaired Student's t‐test. ** *p* < 0.01. c) Western blot analysis of the expression levels of various dCas13 proteins after transfection. Vinculin served as a loading control. dC2C2, catalytically inactive C2C2(Cas13a) from *Leptotrichia shahii*; dRX, catalytically inactive Cas13d from *Ruminococcus flavefaciens XPD3002*; dLW, catalytically inactive Cas13a from *Leptotrichia wadei*; dPGU, catalytically inactive Cas13b from *Porphyromonas gulae*; dPSP, catalytically inactive Cas13b from *Prevotella sp. P5‐125*; dPSPS, truncated catalytically inactive Cas13b from *Prevotella sp. P5‐125*. d) Western blotting was carried out to validate the immunoprecipitation efficiency. GAPDH was used as a negative control. e) Real‐time RT‐PCR measurement of *NEAT1* levels in RNA‒protein complexes immunoprecipitated with beads coated with a Flag antibody. Quantitative data are presented as the means ± SDs based on five independent experiments.

To determine the optimal Cas13 protein for RNA targeting, RNA binding protein immunoprecipitation (RIP) assays were conducted to assess the binding capacity of various catalytically inactive Cas13 orthologs,^[^ ^33^ ^]^ including dCas13a from *Leptotrichia shahii*, dCas13a from *Leptotrichia wadei*, dCas13b from *Porphyromonas gulae*, dCas13b from *Prevotella sp. P5‐125* and dCas13d from *Ruminococcus flavefaciens XPD3002* (**Figure** **1**a). By transfecting wildtype pspCas13b and sgRNAs targeting *NEAT1* plasmids into cells and then measuring the knockdown efficiency of *NEAT1* comparing to Non‐targeting (Non) control, a sgRNA targeting accessible region (the region targeted by sgRNA1) of *NEAT1* was validated (Figure 1b). All sgRNAs of Cas13 orthologs were designed to target the same validated region in *NEAT1*. Western blot confirmed that various catalytically inactive Cas13 orthologs overexpressed at a similar level (Figure 1c). It was found that the truncated dCas13b from *Prevotella sp. P5‐125* (dpspCas13b short version, abbreviated dPSPS) exhibited the highest affinity^[^ ^25^ ^]^ (Figure 1d,e).

### 2.2 Engineering pspCas13b sgRNA for an Alternate Anchoring Position

Previous designs of dCas9‐based transcription activators have shown that in addition to fusing transactivation domains to either the amino or carboxy terminus of the dCas9 protein, engineered sgRNA could tolerate the addition of protein‐interacting RNA aptamers to facilitate the recruitment of effector domains to the Cas9 complex.^[^ ^34^ ^]^ This engineering of sgRNAs has successfully expanded the application of Cas9, including transcription activation, genomic imaging, and prime editing.

**Figure 2:**
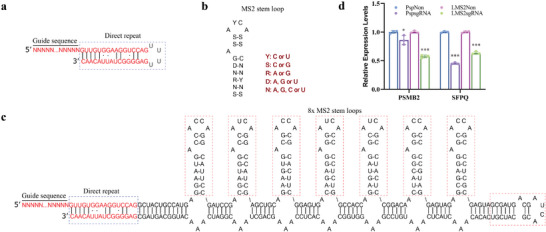
Engineering sgRNA. a) Schematics of the pspCas13b sgRNA. N represents an unspecified nucleoside. b) Consensus sequences of the MS2 stem loop, where Y was replaced with C or U; D with A, G or U; S with C or G; R with G or A; and N with any nucleotide. c) The loop region in the direct repeat of the pspCas13b sgRNA was replaced with eight MS2 stem loops. The MS2 stem loops were adapted from the Sirius‐8xMS2 scaffold to avoid repeating sequences and to optimize RNA secondary structures. d) Real‐time RT‐PCR analysis of the knockdown efficiency of canonical and engineered sgRNAs. Two endogenous genes, *PSMB2* and *SFPQ*, were tested. PspNon, canonical control sgRNA; PspsgRNA, sgRNA targeting PSMB2 or SFPQ; LMS2Non, engineered control sgRNA; LMS2sgRNA, engineered sgRNA targeting PSMB2 or SFPQ. The data were presented as mean ± SDs, *n*  =  3 independent experiments, two‐tailed unpaired Student's *t*‐test. * *p* < 0.05, *** *p* < 0.001.

To explore alternate anchoring positions, we replaced the tetraloop in pspCas13b sgRNA with optimized octets of MS2 aptamers, a strategy inspired by previous engineering designs for CRISPR/Cas9 sgRNA^[^ ^34^, ^35^ ^]^ (**Figure** **2**a–c). Importantly, this modification of sgRNA did not affect the catalytic function of pspCas13b complex. As shown in Figure 2d, pspCas13b effectors with wild‐type or engineered sgRNAs were able to knock down endogenous mRNAs with similar efficiency, suggesting that the tetraloop can tolerate the addition of MS2 aptamers, which may facilitate effector recruitment.

### 2.3 Selection of the Tetraloop of sgRNA as a Tagging Position

A previous study has demonstrated the importance of tag position in dCas9‐based applications. In order to determine the optimal tag positions for dpspCas13b application, we performed an examination assay use BioTAP‐mediated immunoprecipitation of *NEAT1* binding proteins. BioTAP is a small peptide that can be recognized and biotinylated by endogenously expressed biotin protein ligases in eukaryotic cells.^[^ ^36^ ^]^ The biotin‐labeled complex can be easily immunoprecipitated by streptavidin beads, and the tagging efficiency can be assessed by measuring the levels of binding proteins in immunoprecipitated complex.

**Figure 3:**
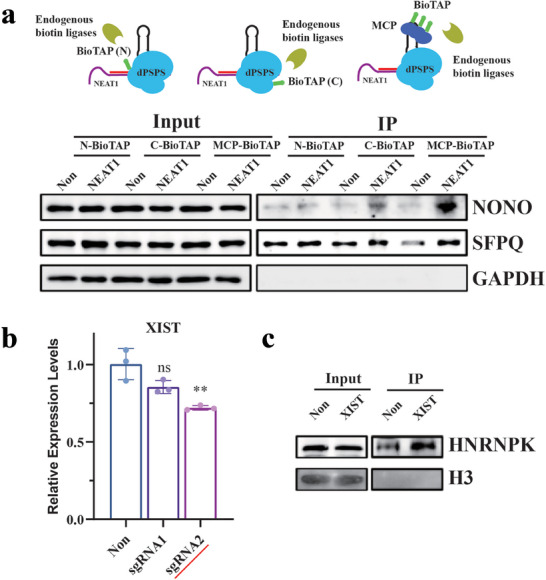
Assessment of tag positions for optimal immunoprecipitation efficiency. a) Schematics of three tagging strategies for BioTAP. BioTAP was tagged in the N‐terminus or C‐terminus of dPSPS or was fused to MCP and tagged via the MS2 stem loops in the sgRNA. By using sgRNAs targeting *NEAT1*, western blotting analysis of the immunoprecipitated RNA–protein complexes showed that tagging via the MS2 stem loops allowed more efficient detection of the *NEAT1* binding proteins NONO and SFPQ. GAPDH was used as a negative control. b) Real‐time RT‐PCR analysis of the targeting efficiency of the *XIST* sgRNAs. sgRNA2 was selected for further study. The data were presented as mean ± SDs, *n*  =  3 independent experiments, two‐tailed unpaired Student's t‐test. ns, not significant, ** *p* < 0.01. c) Tagging via the MS2 stem loops could also be used to detect the binding proteins of *XIST*, HNRNPK. H3 was used as a negative control.

In our experiment, we directly fused BioTAP to either the amino or carboxy terminus of the dpspCas13b. Additionally, we fused BioTAP to MS2‐coat protein (MCP) and assessed the recruitment of BioTAP to the dCas13 complex through MS2 motifs in sgRNAs. Following immunoprecipitation and western blot analysis, we measured the levels of *NEAT1* binding proteins to determine whether tagging in the tetraloop of sgRNA allows more efficient identification of *NEAT1* binding proteins than could dCas13‐BioTAP fusions. As shown in **Figure** **3**a, recruitment of BioTAP via the aptamer in sgRNA proved to be a more efficient method for detecting the *NEAT1* binding proteins NONO and SFPQ. We extended our engineering approach by incorporating MS2 motifs into the tetraloop region of a sgRNA directed against the lncRNA *XIST*, subsequently recruiting BioTAP to the dCas13 complex through this modified sgRNA. This methodology proved equally effective in profiling the protein interactome of *XIST*, as illustrated in Figure 3b,c.

The apparent superiority of the MCP‐BioTAP conjugate suggests that the tetraloop offers a location with diminished steric hindrance, enabling more efficient and natural function when a specific enzyme is tethered at this site. Moreover, the fusion to either the C‐terminal or N‐terminal ends of the dCas13 protein might not confer the enzyme with an optimal range of action, or “labeling radius”. By leveraging multiple MS2 motifs incorporated within the sgRNA, an enzyme moiety attached to the sgRNA itself is more likely to possess an ideal labeling radius. Therefore, we propose that the tetraloop of the sgRNA constitutes an optimal position for tagging purposes, facilitating both the recruitment of auxiliary proteins and the maintenance of their enzymatic activities within a spatially favorable environment.

### 2.4 Development of the sarID Method for Proximal Labeling of lncRNA Interacting Transcripts

In addition to a wide range of CLIP derivative methods, several immunoprecipitation‐free proximal labeling methods have been developed and proven to be efficient alternatives to CLIP. Enzymes such as ADAR,^[^ ^25^ ^]^ APEX2,^[^ ^37^ ^]^ poly(U) polymerase,^[^ ^38^ ^]^ and the more recent APOBEC1^[^ ^39^, ^40^ ^]^ have been used for proximal RNA labeling. By fusing these enzymes to RNA binding proteins (RBPs) and identifying the corresponding tagged transcripts, proximal labeling methods have revolutionized the identification of RBP‐RNA interactome in cells.

**Figure 4:**
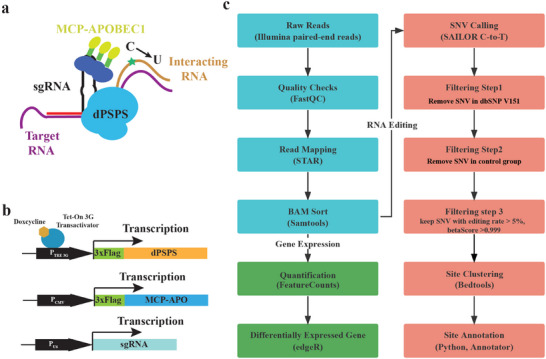
Expression strategy and analysis pipeline of sarID. a) Scheme of the sarID approach. MCP‐APOBEC1 was targeted to sgRNA‐tagged MS2 stem‐loops, mediating the C‐to‐U editing of transcripts proximal to specific lncRNAs. b) Schematics of the expression vectors of dPSPS, MCP‐Apobec1 and sgRNAs. dPSPS expression was driven by a Tet‐On 3G inducible promoter. MCP‐Apobec1 expression was driven by the CMV promoter, and sgRNA expression was driven by the U6 promoter. c) Overview of RNA‐seq and C‐to‐U editing analysis workflow for sarID. Illustration of the computational steps involved in an RNA‐seq experiment to compare the gene expression profiles and to identify the C‐to‐U editing sites in transcripts.

Based on these previous studies, as well as our own findings that truncated dpspCas13b has a higher affinity for target transcripts, that substitutions in the tetraloop region of the sgRNA do not affect Cas13's catalytic function, and that sgRNA is an optimal tagging position, we have developed sarID (**Figure** **4**a). Our aim is to provide a simple and effective method for profiling lncRNA‐RNA interactions in their natural state in living cells, without the need for immunoprecipitation or manipulation of the lncRNA.

To implement sarID in cells, we stably expressed truncated dpspCas13b driven by an inducible promoter and engineered sgRNA using lentivirus. We also stably expressed MCP‐fused APOBEC1, which can be recruited to the Cas13 complex and induce C‐to‐U editing in proximal transcripts^[^ ^39^, ^40^ ^]^ (Figure 4b). By guiding the dCas13 complex to target lncRNA, the recruited APOBEC1 will mediate C‐to‐U editing in the proximal transcripts, thereby labeling the interacting transcripts with C‐to‐U sites. After inducing the expression of dCas13 by adding Doxycycline (Dox) and allowing editing for 48 hours, we purified RNA from the cells and subjected it to RNA‐seq. The SAILOR analysis pipeline was used to identify and quantify the C‐to‐U editing sites in the transcripts,^[^ ^40^ ^]^ following removing editing sites present in control cells and known single nucleotide polymorphisms (SNPs) (Figure 4c). We anticipate that sarID, which allows immunoprecipitation‐free detection of lncRNA‐RNA interactions through standard RNA‐seq, will conveniently and efficiently profile the RNA interactome for specific lncRNAs.

### 2.5 sarID Identifies *NEAT1*‐Interacting Transcripts in Living Cells

**Figure 5:**
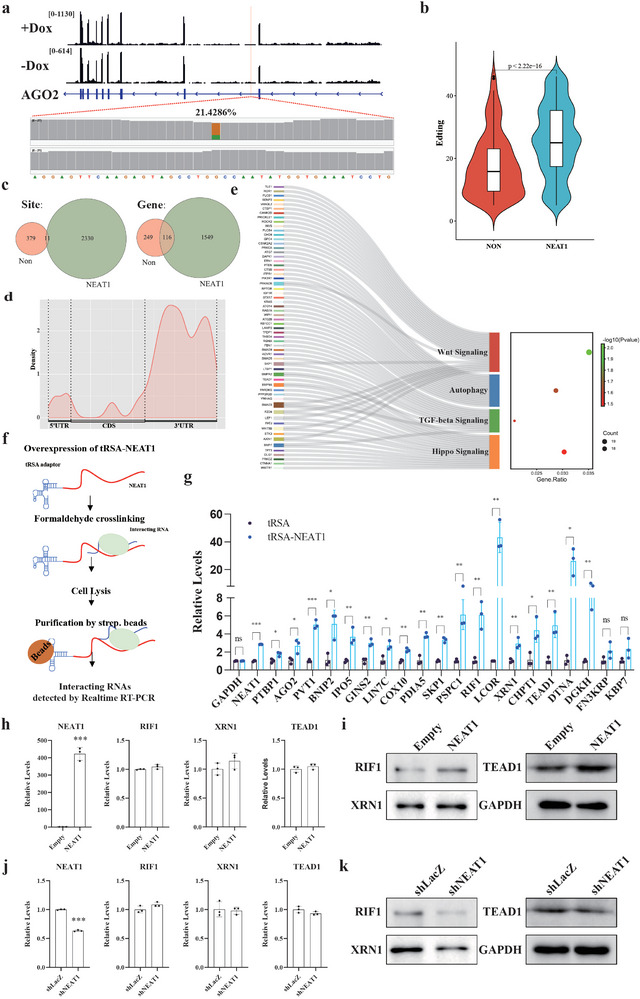
sarID identifies *NEAT1*‐interacting transcripts in living cells. a) Read coverage at the AGO2 locus. C‐to‐U editing was indicated by the color. Brown and green indicate the abundances of C and U sites, respectively. b) Violin plot of C‐to‐U editing rates in the control and *NEAT1* groups. *n*  =  390 and 2341 for control and *NEAT1* respectively; Wilcoxon test. c) Overlap of sites and genes identified in the control and *NEAT1* groups. d) Metagene density plot of the distribution of C‐to‐U editing sites. e) Pathway enrichment analysis of interacting transcripts of *NEAT1*. f) Scheme of the tRSA‐tagged pulldown assay. g) Real‐time RT‐PCR analysis of randomly selected interacting RNAs. GAPDH served as a negative control. The data were presented as mean ± SDs, *n*  =  3 independent experiments, two‐tailed unpaired Student's *t*‐test. ns, not significant, * *p* < 0.05, ** *p* < 0.01, *** *p* < 0.001. h,i) Real‐time RT‐PCR analysis (h) of the RNA levels of and western blot detection (i) of the protein levels of RIF1, XRN1, and TEAD1, when *NEAT1* was overexpressed in 293T cells. j,k) Real‐time RT‐PCR analysis (j) of the RNA levels and western blot detection (k) of the protein levels of RIF1, XRN1, and TEAD1, when *NEAT1* was knocked down in 293T cells. GAPDH was used as loading control in western blot.

To prove the concept, we used sarID to identify the interacting transcripts of *NEAT1*. sarID components with sgRNA targeting *NEAT1* were expressed in HEK293T cells. The protein components of sarID were present in both cytoplasm and nucleus as confirmed by western blot (Figure S1a,b, Supporting Information). RNA‐seq analysis revealed that overexpression of sarID components did not significantly affect the transcriptome (Figure S2a,b, Supporting Information, Tables S1 and S2, Supporting Information). *NEAT1* RNA partners were identified through detection of C‐to‐U editing (**Figure** **5**a). Significantly higher C‐to‐U editing rates were observed in the *NEAT1*‐targeting sgRNA group compared to the control group (Figure 5b; Tables S3 and S4, Supporting Information). Additionally, more editing sites and host genes were identified in the *NEAT1* group, and these editing events occurred mainly in 5′ or 3′ UTRs of genes but were evenly distributed across the chromosomes (Figure 5c,d; Figure S3a, Supporting Information). Despite observing a greater frequency of specific motifs in proximity to editing sites when compared to shuffled sequence controls (Figure S4, Supporting Information), it is noteworthy that the most prominent motifs did not exhibit complementarity to *NEAT1*. This observation leads us to hypothesize that these motifs likely serve as binding sites for additional protein partners. These proteins could potentially facilitate or modulate the interaction between *NEAT1* and its target transcripts. Gene Ontology (GO) and pathway enrichment analyses indicated that cell cycle, autophagy, Wnt signaling, TGFβ signaling, and Hippo signaling, which are known downstream targets of *NEAT1*, were enriched in the identified *NEAT1* RNA partners (Figure 5e; Figure S3b, Supporting Information), suggesting that *NEAT1* may regulate these pathways by interacting with and modulating the related mRNAs instead of by mechanisms revealed previously.^[^ ^41^, ^42^, ^43^, ^44^, ^45^ ^]^

To validate the identified transcripts as bona fide *NEAT1* partners, *NEAT1* tagged with a tRNA scaffold linked to a streptavidin aptamer (tRSA) was overexpressed,^[^ ^13^, ^46^ ^]^ and the corresponding transcripts were detected in the immunoprecipitated RNA‐protein complex using real‐time RT‐PCR^[^ ^46^ ^]^ (Figure 5f). Of the 21 randomly selected targets, 19 showed significant enrichment (Figure 5g), indicating that sarID efficiently identified *NEAT1*‐interacting RNAs.

Upon further examination, we assessed the RNA and protein levels of mRNAs that interact with *NEAT1*, namely RIF1, XRN1, and TEAD1, whose interactions had been previously validated through the tRSA‐RIP assay. Our findings, depicted in Figure 5h,i, revealed that while the mRNA levels of these genes remained unchanged, their protein levels exhibited a significant increase when *NEAT1* was overexpressed in 293T cells. Conversely, as shown in Figure 5j,k, the protein levels of these genes decreased when *NEAT1* was knocked down using shRNA (short hairpin RNA), yet their mRNA levels remained stable. These observations collectively suggest that the interaction between *NEAT1* and its associated mRNAs plays a pivotal role in the post‐transcriptional regulation of gene expression.

### 2.6 sarID Identifies RNA Partners of the lncRNA *XIST* and *MALAT1* in Living Cells

**Figure 6:**
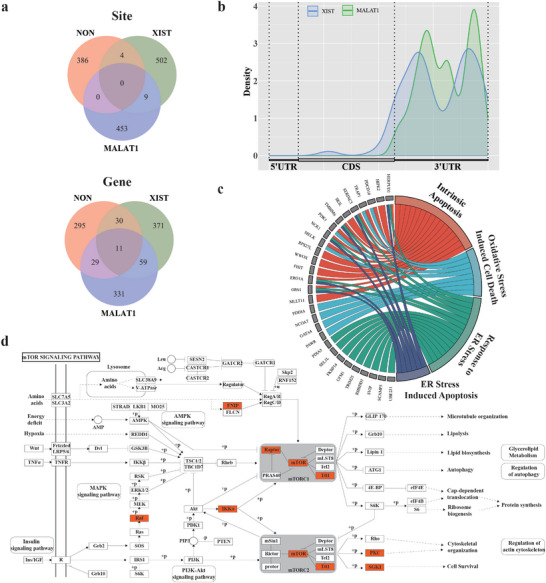
sarID identifies RNA partners of the lncRNA *XIST* and *MALAT1* in living cells. a) Overlap of sites and genes identified in the control, *XIST* and *MALAT1* groups. b) Metagene density plot of the distribution of C‐to‐U editing sites. plots for *XIST* and *MALAT1* are shown in blue and green, respectively. c) Chord diagram of significant GO terms related to cell death with enrichment of *XIST*‐interacting genes. d) KEGG pathway map for the mTOR signaling pathway. Red Indicates *MALAT1* interacting genes.

To generalize the application of sarID, we designed sgRNAs to target *XIST* and *MALAT1* (Figure 3b; Figures S1b and S5a, Supporting Information). After applying sarID in HEK293T cells and confirming that the transcriptome was not affected (Figure S2c,d, Tables S5 and S6, Supporting Information), we identified 515 C‐to‐U editing events corresponding to 471 genes for *XIST* and 462 C‐to‐U editing events corresponding to 430 genes for *MALAT1* (**Figure** **6**a; Tables S7 and S8, Supporting Information). The editing sites identified were located predominantly in the 3′ UTR of genes and were evenly distributed along chromosomes (Figure 6b; Figure S6a,b, Supporting Information). Among the interacting genes identified for *XIST* and *MALAT1*, only 65/471 and 41/430 could be detected by genome‐wide RNA‐RNA interaction approaches (for *NEAT1*, only 59/1665 could be similarly detected),^[^ ^47^ ^]^ suggesting that sarID was more sensitive than existing genome‐wide RNA‐RNA identification methods for specific lncRNAs. Notably, pathway enrichment analysis revealed that stress‐induced cell death pathways were enriched in *XIST* partners, consistent with the role of *XIST* in the regulation of cell death in tumors^[^ ^48^ ^]^ (Figure 6c). Additionally, *MALAT1* regulates mTOR signaling,^[^ ^49^ ^]^ and we successfully identified enrichment of mTOR signaling genes among the *MALAT1* partners (Figure 6d). These results demonstrate the applicability of sarID for identifying RNA partners of lncRNAs.

### 2.7 sarID Identifies RNA Partners of the lncRNA *NBR2* and *DANCR* with High Reproducibility

**Figure 7:**
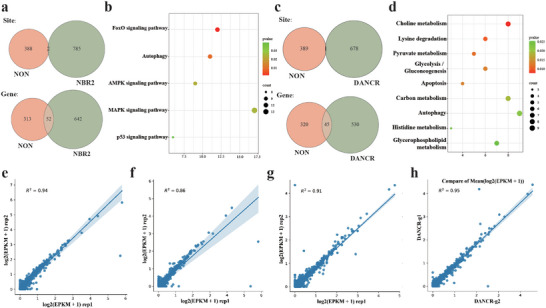
sarID identifies interacting RNAs of the lncRNA *NBR2* and *DANCR*. a) Overlap of sites and genes identified in the control and *NBR2* groups. b) Pathway enrichment analysis of interacting transcripts of *NBR2*. c) Overlap of sites and genes identified in the control and *DANCR* groups. d) Pathway enrichment analysis of interacting transcripts of *DANCR*. e–g) correlations of EPKM between repeats of sgRNA targeting *NBR2* (e), sgRNA1 (f), and sgRNA2 (g) targeting *DANCR* respectively. h) correlations for EPKM between sgRNA1 and sgRNA2 targeting *DANCR*.

The sarID method has proven its capability and reliability in identifying RNA partners of lncRNAs, initially demonstrating success with abundant and nuclear lncRNAs such as *NEAT1*, *XIST*, and *MALAT1*. To further validate the scope and consistency of sarID, particularly with regard to cytoplasmic and lower‐abundance lncRNAs, we applied it to *NBR2* and *DANCR* (Figures S1c,d and S5b,c, Supporting Information). Our preliminary checks confirmed that their transcriptomes were unaffected by the sarID procedure (Figure S2e,f, Tables S9 and S10, Supporting Information). For *NBR2*, we identified 787 C‐to‐U editing events across 694 genes, and for *DANCR*, 679 editing events across 575 genes (**Figure** **7**a,c; Tables S11 and S12, Supporting Information). Given the minimal overlap with non‐target control group, these editing sites and their associated transcripts are strong candidates for being authentic targets of *NBR2* and *DANCR*. Pathway enrichment analyses of the identified partners revealed intriguing insights. For instance, energy stress‐related pathways, including FOXO, AMPK, and autophagy, were prominently enriched among *NBR2*'s partners, aligning with *NBR2*'s known role in AMPK‐mediated metabolic checkpoints^[^ ^50^ ^]^ (Figure 7b). Similarly, multiple metabolic pathways were found enriched among *DANCR*'s partners, hinting at *DANCR*'s potential involvement in cellular metabolism regulation, a finding consistent with previous studies^[^ ^51^ ^]^ (Figure 7d).

To rigorously test the reproducibility of the sarID method, we performed replicate experiments and analyzed as a previous study.^[^ ^40^ ^]^ The number of edited reads per kilobase of transcripts per million mapped reads (EPKM) showed high reproducibility between replicates (R^2^ = 0.94 for *NBR2*, R^2^ = 0.86 and R^2^ = 0.91 for two different sgRNAs targeting *DANCR*, respectively; Figure 7e–g). Notably, the reproducibility was maintained even when different sgRNAs targeting *DANCR* were used (Figure 7h). These results collectively underscore the robustness and reliability of the sarID approach in identifying RNA partners of lncRNAs, even those that are less abundant or located in the cytoplasm.

## 3 Discussion

In this study, we designed and validated the sarID method for identifying the transcript partners of specific lncRNAs. This was achieved by screening optimal dCas13 effector and tag positions in the dCas13‐sgRNA complex, and coupling it with the APOBEC1 RNA labeling approach. The sarID method allows for the mapping of lncRNA‐RNA interactions in the native cellular environment, without any genomic manipulation of the genes encoding the RNA of interest. Additionally, it does not require cross‐linking or immunoprecipitation.

By applying the sarID method to lncRNAs *NEAT1*, *XIST*, *MALAT1*, *NBR2*, and *DANCR*, we were able to identify hundreds of previously unknown transcript partners. Most of these partners were found to be mRNAs, suggesting a new gene expression paradigm involving lncRNA‐mRNA interactions beyond their known functions as protein scaffolds, miRNA sponges, or guides for epigenetic modulators. It is suspected that lncRNAs may bind to mRNAs to regulate their subcellular locations, stability, RNA modifications, or translation capacity. Further studies are needed to elucidate the underlying molecular mechanisms.

Comparing the results of sarID to current genome‐wide RNA‐RNA interaction methods, we found that sarID identified more transcript partners for *NEAT1*, *XIST*, and *MALAT1*.^[^ ^47^ ^]^ This suggests that sarID is more sensitive in identifying the RNA interactome of specific lncRNAs compared to currently available approaches. Although sarID has currently only been applied to lncRNAs, the same method can be easily adapted without modification to profile the RNA interactome of other RNAs, such as circRNAs (by targeting the circRNA‐specific back‐splice junction), snoRNAs, and even mRNAs. It is hoped that sarID can help profile the RNA interactome of these RNAs.

In addition to identifying RNA interacting partners, it is critical to identify the binding proteins of lncRNAs in order to understand their function and mechanism. Previous studies have shown that directly fusing proximal labeling enzymes, such as PafA,^[^ ^28^ ^]^ APEX2,^[^ ^29^ ^]^ and BASU,^[^ ^30^ ^]^ to dCas13 leads to the proximal labeling of binding proteins for specific lncRNAs. However, our results suggest that the tetraloop of sgRNA is an optimal tagging position. It would be interesting to investigate whether tagging these enzymes to engineered sgRNAs would result in more efficient labeling of proximal proteins.

Given the differences in length of RNA, it is theoretically not possible for one sgRNA to represent the entire pattern of a whole RNA. However, sarID offers a potential tool for studying RNA‐RNA interactions at specific positions on target RNAs. Additionally, the spatial structural uncertainty of the target RNA is often largely unknown. Utilizing a more potent sgRNA may significantly enhance the identification of a broader spectrum of interacting transcripts, thus not only boosts the sensitivity and specificity of the technique but also expands its capacity to uncover previously uncharted RNA partnerships.

In addition to APOBEC1, APEX2,^[^ ^37^ ^]^ ADAR,^[^ ^25^ ^]^ and poly(U) polymerase^[^ ^38^ ^]^ have also been successfully used as proximal RNA labeling enzymes. These enzymes have varying labeling radius, kinetics, and chemistry. Substituting the labeling enzyme of sarID with different enzymes would expand the toolbox for investigating the landscape of a wide range of RNA interactions within living cells. We anticipate the development of a growing repertoire of RNA‐centered proximity labeling tools to further enhance our analysis of the cellular RNA‐RNA interactome.

## 4 Experimental Section

### Plasmid Cloning

For the overexpression of various dCas13 orthologs, PCR products from corresponding plasmids (dC2C2, Addgene #79150 with point mutations generated as described in previous study;^[^ ^52^ ^]^ dCasRx, Addgene# 153209; dCasLw, Addgene#91905; dPguCas13, pHAGE‐dPguCas13b; dPspCas13, pHAGE‐dPspCas13b; dPspCas13 short, Addgene# 103871) were subcloned to pCDH‐3xFlag‐puro plasmids. sgRNAs targeting *NEAT1*, *MALAT1*, *XIST*, *PSMB2*, and *SFPQ* were designed online^[^ ^53^ ^]^ (https://cas13design.nygenome.org/). shRNA, sgRNA, or engineered sgRNA backbones were synthesized by Tsingke Biotechnology Co. Ltd. (Beijing, China) and cloned to pLKO.1 vector (Addgene#10878). Target sequences were annealed and cloned into corresponding sgRNA expression vectors. For overexpressing tRSA tagged *NEAT1*, the tRSA sequence amplified from pcDNA3‐tRSA (Addgene# 32200) was subcloned between the XbaI and NotI sites into the pCDH‐puro vector. *NEAT1* sequence was inserted into the EcoRV site of the pCDH‐tRSA vector as previous.^[^ ^13^ ^]^ dPspCas13‐BioTAP, BioTAP‐dPspCas13, V5 tagged dPSPS were synthesized and were subcloned into pCDH‐Blastcidin plasmid. MCP‐BioTAP and MCP‐APOBEC1 were synthesized and were subcloned into pCHD‐Hygromycin vector. 3xFlag tagged dPSPS were synthesized and were subcloned into pCDH‐TRE3G‐EF1‐Teton3G‐P2A‐Blastcidin vector. The sequences of sgRNAs used in this study are listed in Table S13 (Supporting Information).

### Cell Culture and Stable Cells

HEK293T cells were obtained from ATCC and were cultured with Dulbecco's modified Eagle's medium (DMEM, Gibco) adding 10% Fetal Bovine Serum (FBS, Gibco) and 1% 100 U/ml penicillin/streptomycin (Keygen BioTECH) at 37 °C cell incubator containing 5% CO_2_. To generate stable cells, Lentiviruses were produced in HEK293T cells by transfecting the expression plasmids with packaging plasmids (psPAX.2: pMD2.G = 3:1) and a transfection solution (polyethyleneimine; PEI). After incubation for 48 h, the lentivirus media were harvested by centrifuging at 3500 rpm for 5 min and subsequently filtered using 0.45 µm filter. The Cells were then infected with lentivirus and selected using puromycin (InvivoGen) or blasticidin (InvivoGen).

### Subcellular Fraction

Subcellular fractionation was performed as previously described.^[^ ^54^, ^55^ ^]^ Cells were collected and washed with PBS. Cell pellets were lysed in buffer I (20 mm HEPES, 10 mm KCl, 2 mm MgCl_2_, and 0.5% NP40), and the supernatants were collected for cytoplasmic lysis. Pellets were further lysed in buffer II (0.5 m NaCl, 20 mm HEPES, 10 mm KCl, 2 mm MgCl_2_, and 0.5% NP40) and supernatants were collected for nuclear lysis. Cytoplasmic and nuclear fractions were separated for protein extraction, and western blotting.

### Protein Extraction and Western Blotting

Cells were harvested and lysed by RIPA lysis buffer with protease inhibitor (Roche) and phosphatase inhibitor (Roche) for 30 min at 4 °C. Protein was quantified using a BCA kit (Keygen BioTECH) according to the manufacturer's instructions. For western blotting, 30 µg protein per well was separated by SDS‐PAGE, and transferred to nitrocellulose membrane (Millipore). Then, membranes were blocked with 5% bovine serum albumin (BSA) for 1 h, then incubated with primary antibodies overnight at 4 °C. Horseradish peroxidase‐conjugated secondary antibodies were used to detect primary antibodies. Immunoreactivity was determined using ECL method and imaged using a Bio‐Rad multiple‐function imager.

Primary antibodies used: GAPDH (Cell Signaling Technology Cat#2118), vinculin (Proteintech Cat#66305‐1‐Ig), NONO (Proteintech Cat#11058‐1‐AP), SFPQ (Proteintech Cat#15585‐1‐AP), Flag (Sigma, Cat#F1804), HNRNPK (Proteintech Cat#11426‐1‐AP), TEAD1 (Proteintech Cat#13283‐1‐AP), XRN1 (Bethyl Laboratories Cat# A300‐443A), RIF1 (Bethyl Laboratories Cat# A301‐102A), Lamin A/C (Cell Signaling Technology Cat# 2032).

Secondary antibodies used: anti‐mouse IgG, HRP‐linked Antibody (Cell Signaling Technology Cat#7076); anti‐rabbit IgG, HRP‐linked Antibody (Cell Signaling Technology Cat#7074).

### Flag Tagged RIP

sgRNA and dCas13 vectors were transfected into HEK293T cells by using PEI (Polysciences, Cat#24765). 48 hours after transfection, cells were washed with PBS and crosslinked with fresh 0.75% formaldehyde followed by 1.25 m glycine quenching. The cross‐linked cells were resuspended with RIPA lysis buffer supplemented with protease inhibitor (Roche), phosphatase inhibitor (Roche), and nuclease inhibitor (Accurate Biotechnology, AG), and allowed to sonicate for 20 min with a 5s on / 5s off cycle at 80% power on a sonicator (SCIENTZ 08‐III) at 4 °C. Insoluble debris was cleared by centrifugation at 14 000 g for 10 min at 4 °C, and supernatant was then incubated with 30 µL anti‐Flag magnetic beads (Invitrogen) overnight on a rotator at 4 °C. The beads were washed three times with wash buffer (2 × SSC, 0.5% SDS). The beads were divided into two portions (40% for RNA and 60% for protein analysis). Proteins were analyzed using western blotting. To purify RNA, beads were resuspended in 100 µL 1 × DNase buffer supplemented with DNase I (AG) and nuclease inhibitor (AG) followed by incubation at 37 °C for 30 min with 1200 rpm rotation. Beads were digested by the addition of Protease K (AG) at 60 °C for 30 min with 1200 rpm rotation. Next, the MicroElute RNA clean‐up kit (Omega) was used to purify RNA as described in the manufacturer's instructions.

### tRSA tagged RIP

tRSA‐*NEAT1* and tRSA control vectors were transfected into HEK293T cells by using PEI (Polysciences, Cat#24765) for 48 hours. RIP was performed as Flag tagged RIP except that streptavidin magnetic beads C1 (Invitrogen) were used instead of anti‐Flag magnetic beads.

### Real‐Time RT‐PCR

Real‐time RT‐PCR was performed using the SYBR qPCR Master Mix (Vazyme). All experiments were performed according to the manufacturer's instructions. Relative gene expression levels were calculated by using 2^−ΔΔCt^ method. The primers used are listed in Table S13 (Supporting Information).

### Transcriptome Analysis

Stable cells were treated with 1µg ml^−1^ of Doxycline for a duration of 48 hours. Post‐treatment, paired‐end reads (2 × 150 nucleotides) were employed to sequence the transcriptome. The fastp (v0.22.0) tool was utilized to eliminate adapter sequences and low‐quality reads. For read alignment, the STAR 2‐pass mode (v2.7.8a) was applied to map the reads onto the hg38 reference genome. Subsequently, samtools was used to remove unaligned reads and sort the aligned reads based on their coordinates. Following that, featureCounts (v2.0.3) was used to quantify gene‐level expression, and edgeR was employed to conduct differential analysis for non‐repeated samples. During the differential analysis, genes with a minimum count value of 5 and those with total count values <15 in the control and experimental groups were filtered out. The dispersion was calculated using the generalized linear model (glmLRT) to determine the level of expression difference for each gene. Genes with an FDR<0.05 and |logFC|>1 were considered as differentially expressed genes.

### Identification C‐To‐U Editing Sites

In the study, further analysis was conducted on the sorted BAM files. SAILOR (1.2.0) was utilized to identify C‐to‐U editing sites, employing the reference genome hg38 and including the default parameter “ct:true”. To ensure precision, the study excluded known SNPs (dbSNP, v151) and sites with a betaScore <0.5. Subsequently, annotator (v0.14.2) was employed to provide annotations for the identified sites. Bedtools were then utilized in order to filter out SNP sites present in the control group that corresponded to the experimental group. This filtering process was based on multiple criteria, including betaScore > 0.99, mutation reads count > 1, edit rate > 0.05, and total reads count > 10. EPKM values for genes were calculated as the previous study.^[^ ^40^ ^]^ GO and pathway enrichment analyses were performed using online tools (https://www.bioinformatics.com.cn/).

### Statistical Analysis

All data were obtained from at least three independent experiments. All statistical graphs were shown as mean ± standard deviation (s.d.). Statistical differences were performed using a two‐tailed t‐test in GraphPad Prism 8.0. A *p*‐value <0.05 is statistically significant. Sample sizes were selected based on general practices in the field. No statistical methods were used to determine the sample size. The investigators were not blinded to the allocation during the experiments or outcome assessment.

## Conflict of Interest

The authors declare no conflict of interest.

## Author Contributions

Z.‐D. X. and Q. Z. conceived the project. L.‐T.D., S.‐J.X., W.‐Y.X., and X.‐X.L. carried out experiments. H.‐H.Z. and J.‐B.L. performed data analysis. Z.‐D.X., Q.Z., and L.‐T.D. wrote the manuscript.

## Supporting information



Supporting Information

Supporting Information

Supporting Information

Supporting Information

Supporting Information

Supporting Information

Supporting Information

Supporting Information

Supporting Information

Supporting Information

Supporting Information

Supporting Information

Supporting Information

Supporting Information

## Data Availability

The raw data were deposited in the NCBI BioProject database under the accession number PRJNA979935.
