# Supplementary material for: CRISPR/Cas13 sgRNA‐Mediated RNA–RNA Interaction Mapping in Live Cells with APOBEC RNA Editing
Source: Adv Sci (Weinh). 2024 Oct 11;11(45):2409004. doi: 10.1002/advs.202409004 (PMC11615753; doi:10.1002/advs.202409004)
Supplement: Supplementary file 1 — Supporting Information [file ADVS-11-2409004-s006.docx]

**
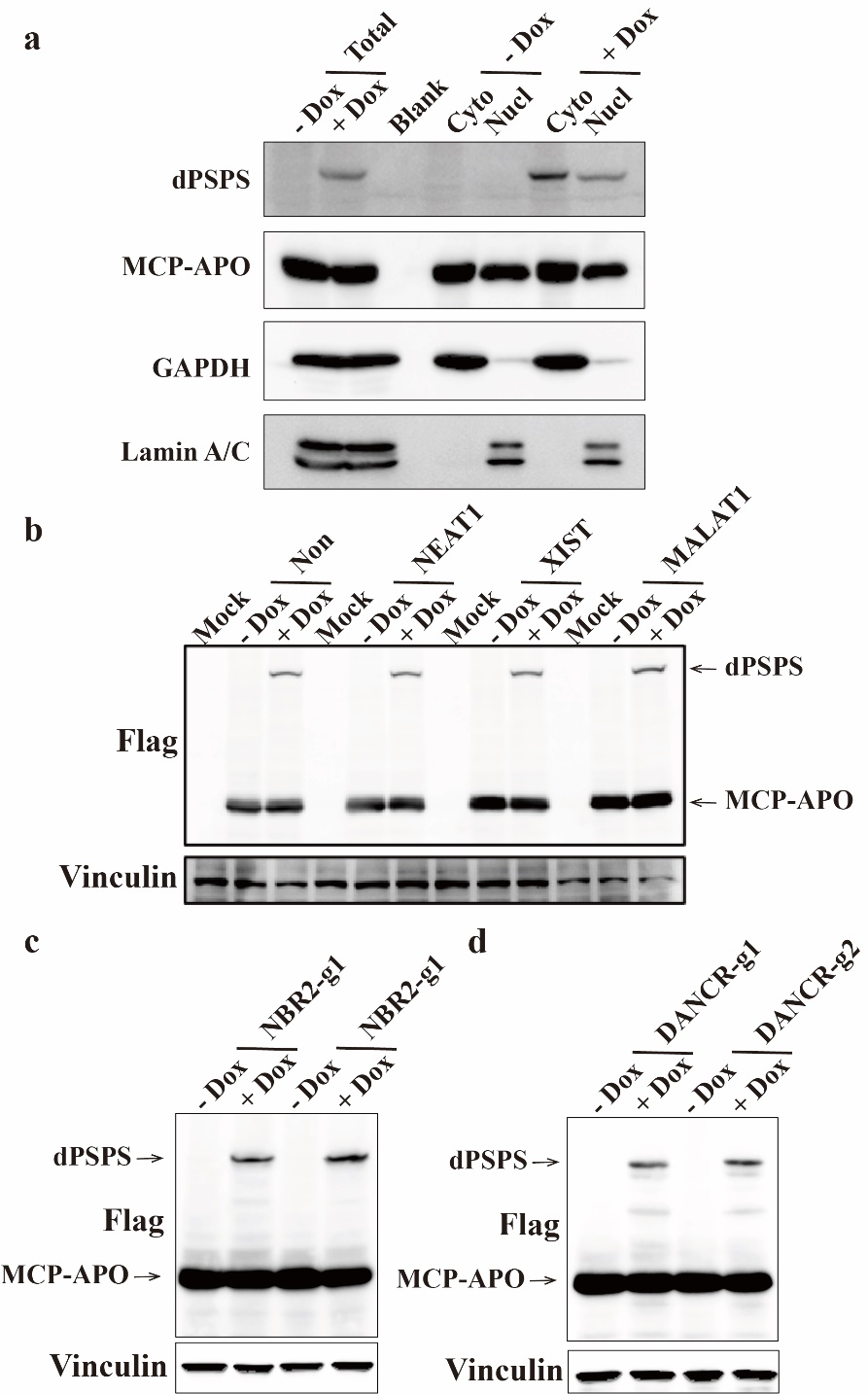
**

**Supplementary Figure 1. Expression detection of sarID components. a**, Cells were cultured with and without doxycycline for 24 hours, then subjected to nucleus (nucl)/cytoplasm (cyto) fractionation analysis and western blotting. GAPDH and Lamin A/C served as markers of cytoplasm and nucleus in western blotting. **b,** Western blotting analysis of the expression of dPSPS and MCP-APOBEC1 in control, *NEAT1*-targeting, *XIST*-targeting and *MALAT1*-targeting stable cells. **c,** Western blotting analysis of the expression of dPSPS and MCP-APOBEC1 in two replicates of *NBR2*-targeting stable cells. **d,** Western blotting analysis of the expression of dPSPS and MCP-APOBEC1 in two sgRNAs targeting *DANCR* stable cells

**
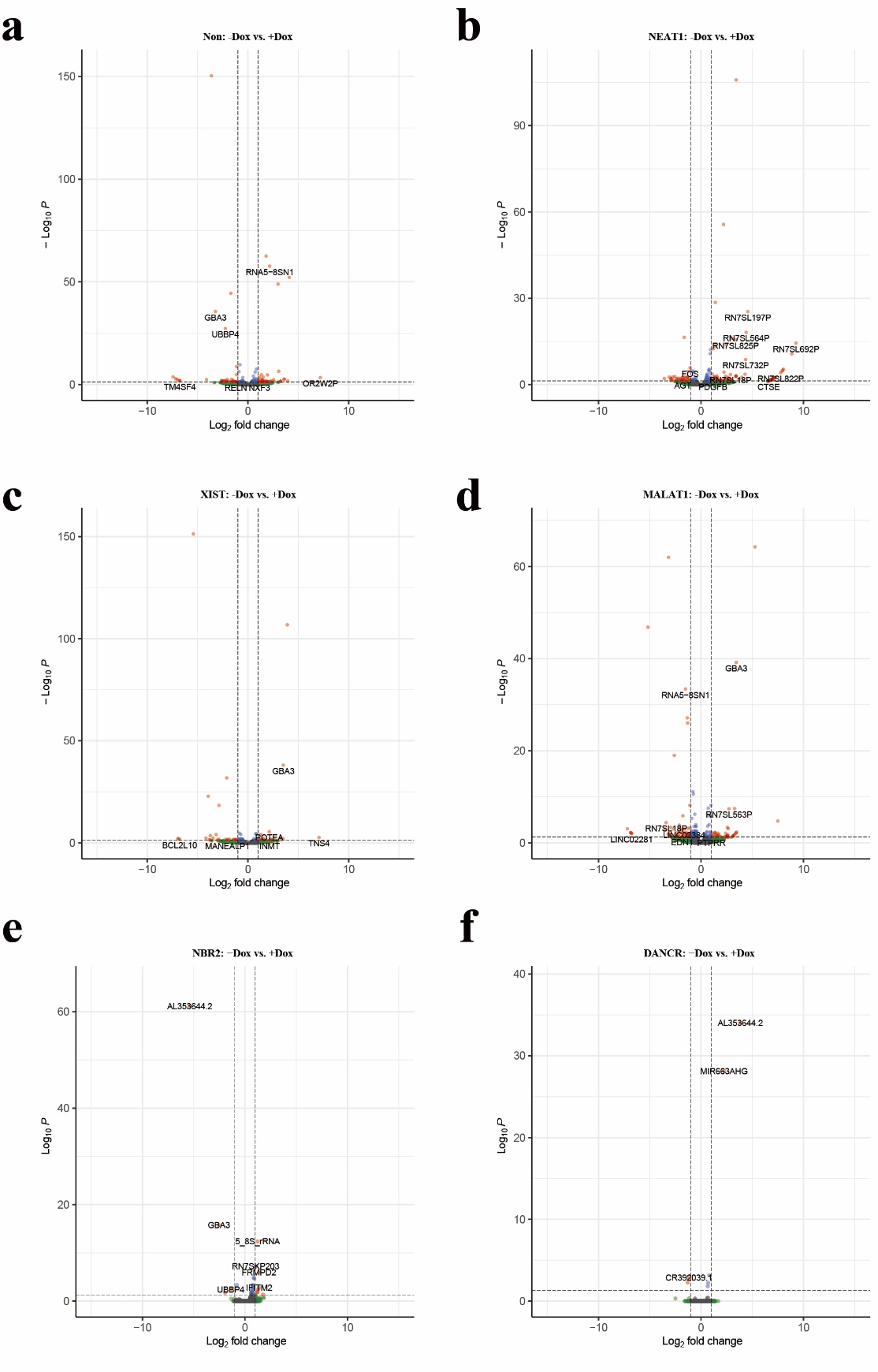
**

**Supplementary Figure 2. Expressing sarID components did not alter the transcriptome. a-f**, Volcano plots of all differentially expressed genes in the presence and absence of doxycycline (Dox) between cells with stable expression of the control sgRNA (**a**) or sgRNAs targeting *NEAT1* (**b**), *XIST* (**c**), *MALAT1* (**d**), *NBR2* (**e**) and *DANCR* (**f**).

**
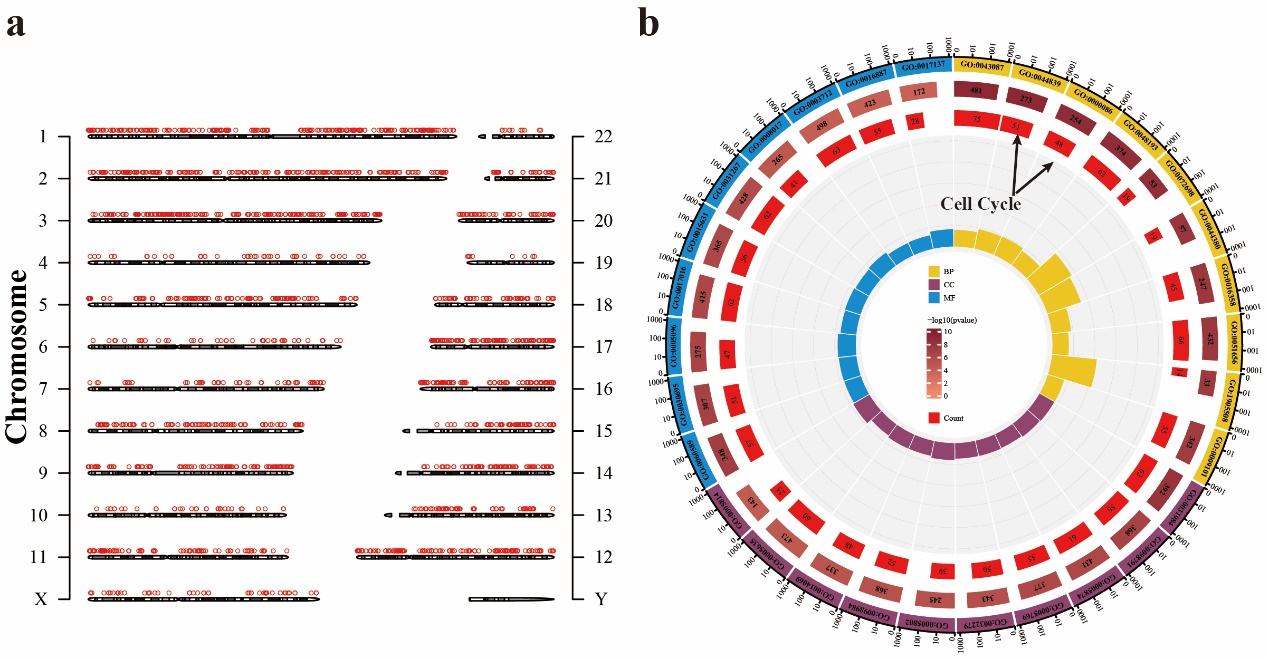
**

**Supplementary Figure 3. Characterization of C-to-U editing sites for *NEAT1*. a**, Genomic distribution of C-to-U editing sites. **b**, Selected enriched GO terms. The first circle indicates the selected GO terms. Cell cycle-related GO terms are highlighted. The second, third and fourth circles indicate the number of genes in the genomic background, the number of enriched genes and the enrichment factor for each GO term, respectively.

**
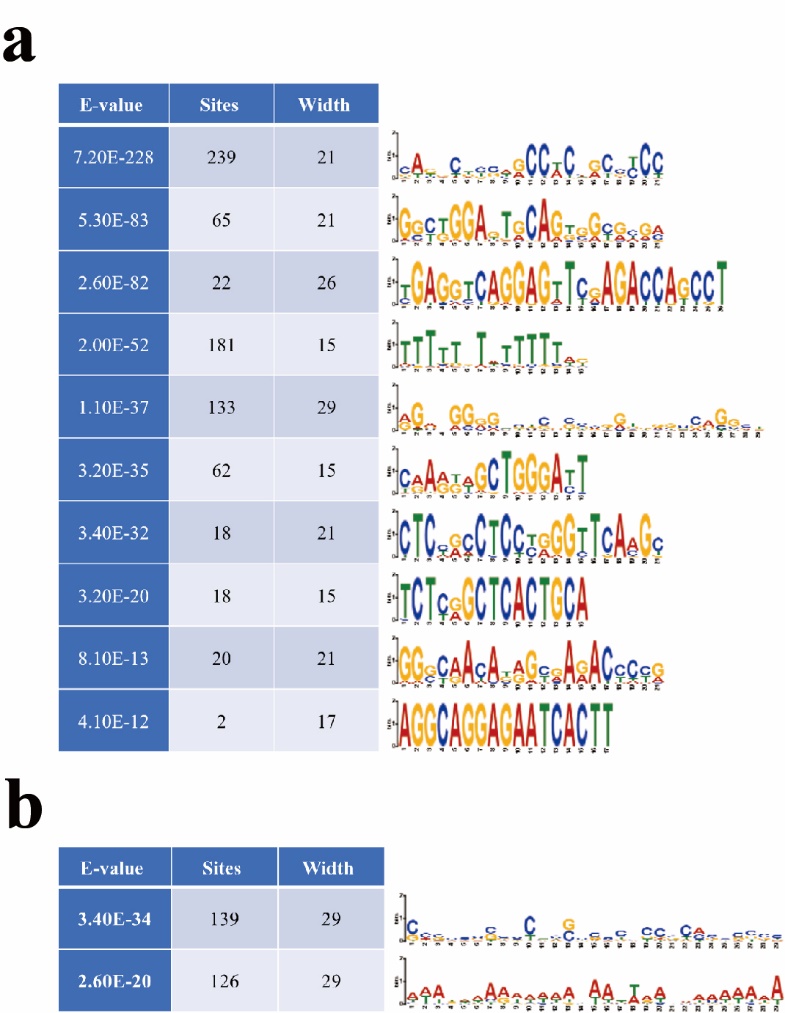
**

**Supplementary Figure 4. Motifs surrounding editing sites of *NEAT1* interacting transcripts. a**, Top 10 motifs discovered by MEME surrounding editing sites of *NEAT1* interacting transcripts. **b**, Motifs discovered in shuffled sequences.

**
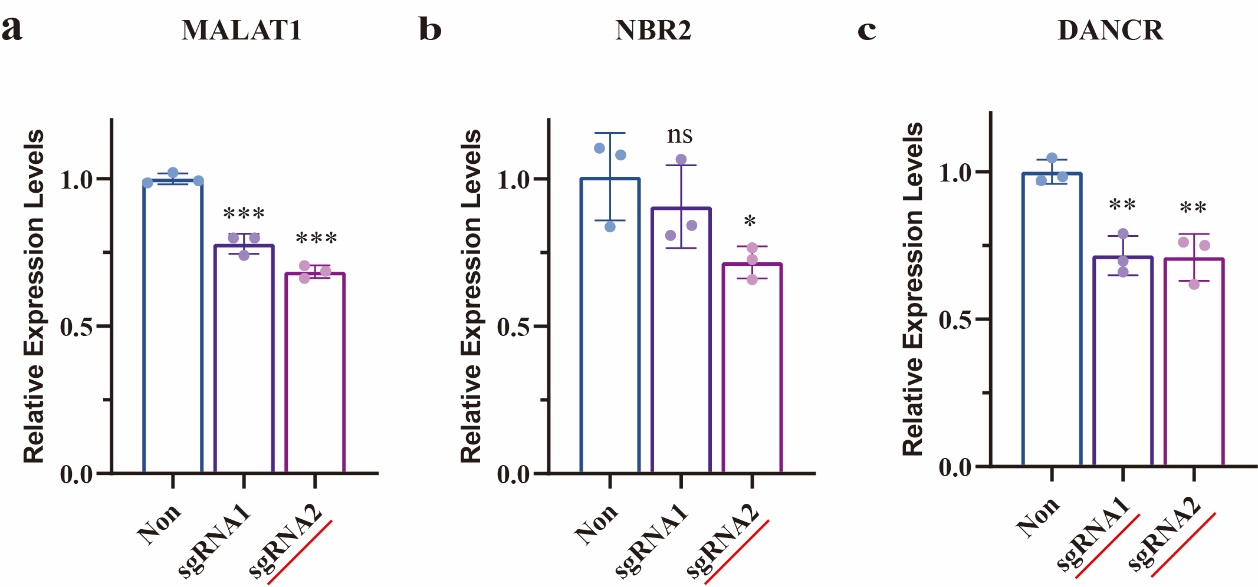
**

**Supplementary Figure 5. Real-time RT-PCR analysis of the targeting efficiency of sgRNAs. a-c,** For *MALAT1*(**a**), *NBR2* (**b**) and *DANCR* (**c**), two sgRNAs for each lncRNA were tested. sgRNAs used in further study were underlined**.** The data were presented as mean ± SDs, n = 3 independent experiments, two-tailed unpaired Student’s t-test. ns, not significant, * P < 0.05, ** P < 0.01, *** P < 0.001.

**
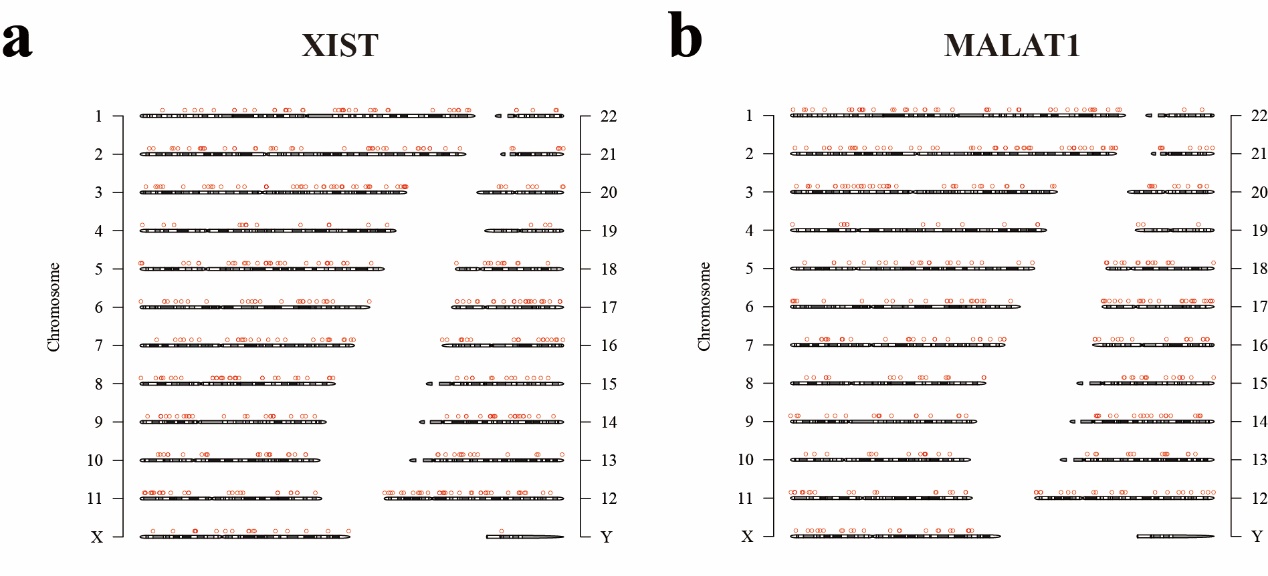
**

**Supplementary Figure 6. Genomic distribution of C-to-U editing sites. a**, **b**, Genomic distribution of C-to-U editing sites for *XIST* (**a**) and *MALAT1* (**b**), respectively.
